# Biodegradation Process of PSF-PUR Blend Hollow Fiber Membranes Using *Escherichia coli* Bacteria—Evaluation of Changes in Properties and Porosity

**DOI:** 10.3390/polym13081311

**Published:** 2021-04-16

**Authors:** Wioleta Sikorska, Małgorzata Milner-Krawczyk, Monika Wasyłeczko, Cezary Wojciechowski, Andrzej Chwojnowski

**Affiliations:** 1 Nałęcz Institute of Biocybernetics and Biomedical Engineering, Polish Academy of Sciences, Trojdena 4 Str., 02-109 Warsaw, Poland; mwasyleczko@ibib.waw.pl (M.W.); cwojciechowski@ibib.waw.pl (C.W.); achwojnowski@ibib.waw.pl (A.C.); 2 Faculty of Chemistry, Warsaw University of Technology, Noakowskiego 3 Str., 00-644 Warsaw, Poland; mmilnerkrawczyk@ch.pw.edu.pl

**Keywords:** partly degradable hollow fiber membranes, biodegradation process, PSF-PUR membranes, PUR degradation, *E. coli*

## Abstract

This work was focused on biodegradation with *Escherichia coli* bacteria studies of PSF-PUR blend semipermeable hollow fiber membranes that possibly can undergo a partial degradation process. Hollow fiber membranes were obtained from polysulfone (PSF) and polyurethane (PUR) containing ester bonds in the polymer chain in various weight ratios using two solvents: *N*,*N*-Dimethylmethanamide (DMF) or *N*-Methylpyrrolidone (NMP). The membranes that underwent the biodegradation process were tested for changes in the ultrafiltration coefficient (UFC), retention and cut-off point. Moreover, the membranes were subjected to scanning electron microscopy (SEM), MeMoExplorer^TM^ Software and Fourier-transform infrared spectroscopy (FT-IR) analysis. The influence of *E. coli* and its metabolites has been proven by the increase in UFC after biodegradation and changes in the selectivity and porosity of individual membranes after the biodegradation process.

## 1. Introduction 

Polysulfone (PSF) is characterized by chemical inertness in the entire pH range, compressive strength, thermal stability (150–170 °C) and mechanical strength (breaking, bending, twisting). This makes it one of the most popular synthetic polymer materials used in the production of membranes [1,2,3]. PSF membranes are widely used in microfiltration (MF) [4] and ultrafiltration (UF) [5], gas separation (GS) [6,7], pervaporation [8], hemodialysis [9,10,11], plasma separators [12], membrane oxygenators [13], cell cultures [14]. The market share of PSF membranes is constantly growing, e.g., it is the most frequently used membrane in the CO_2_/CH_4_ separation process due to its low price, chemical stability and mechanical strength [1,2,3,6,7].

Morphology, structure, chemical and thermal properties are all important features that should be carefully studied to improve materials and ensure better performance. The combination of all these properties determines the suitability of the membrane. A key factor in the development and application of polymer membranes is the control of their polymer morphology. Porosity is the main factor influencing the morphological properties of the membrane, changing the filtration efficiency and the fouling effect [1,2,3,15]. 

Polymer blends have become a simple, constructive and flexible approach to improve membrane properties. Obtaining membranes from a polymer blend requires compatibility concerning the basic properties of the polymers. They should have similar physical, chemical properties, which will result in their proper mixing in the right solvent. The correct selection and combination of the polymer components in the correct proportions can lead to a membrane with favorable anti-fouling properties [3,16]. For example, it has been found that the presence of hydrophilic polyethylene glycol (PEG) 600 Da and its concentration plays a key role in altering membrane properties such as porosity and pore size. The addition of PEG to the membrane dope mixture improved the efficiency of the separation of proteins and metal ions and increased the pure water flux (PWF) [17,18,19]. There are multiple examples of use polyvinylpyrrolidone (PVP) or PEG as a pore precursor [15,17,20,21,22,23,24,25,26,27]. In recent years polymer blending is a widely used technique for modifying cheap materials in order to improve their properties. Polymer blends can be classified as homogeneous/miscible and heterogeneous/incompatible. Homogeneous mixtures are relatively rare because the Gibbs free mixing energy is positive due to the change in entropy as a consequence of mixing high molecular weight (MW) polymers [16]. The force responsible for miscibility is that there is a specific interaction between the polymers. There are certain mixtures described as compatible, consisting of, for example, polybenzimidazoles and aromatic polyimide (PI), PI mixed with polyethersulfone PES, and PSF or PSF with PES [28]. 

It is also worth paying attention to membranes made of polymer mixtures, where one of the components is a partially degradable polymer (subject to e.g., hydrolysis). Examples of such membranes are blends of two types of hydrolyzable polymers: polyurethane(PUR) and cellulose acetate(CA). The obtained membranes are characterized by SEM, determination of the ultrafiltration coefficient (UFC), measurements of mass and retention coefficients, and determination of molecular weight cut-off MWCO both after membrane preparation and after hydrolysis with 2 M NaOH solution. It was found that the treatment with a 2 M NaOH solution caused partial removal of CA or PUR, changes in membranes’ structure and increase of membranes permeability without changing the cut-off point [29,30].

In addition to the chemical degradation of membranes, various methods of biodegradation are described in the literature. Controlled biodegradation of membranes aims to improve their transport and separation properties, such as increasing hydraulic permeability and porosity, and increasing the size of the pores. It is worth attention to study biodegradation of membranes due to various possibilities of using membranes in bioprocesses. There are microorganisms in bioprocesses that can cause the natural biodegradation of membranes, therefore membranes should be tested for biodegradation. For example the in vitro biodegradation of PELA electrospun membranes containing Proteinase K (PELA-P) was tested in a Tris-HCl buffer solution at pH 8.6 and 37 °C in comparison to electrospun membranes without proteinase K. During the biodegradation, weight loss, water absorption, incubation buffer pH, capillary morphology and thermal properties were verified [31]. Arabic gum (AG) harvested from Acacia Senegaltree was used with PVA to prepare a range of biodegradable membranes. Bioplastic membranes were degraded by selected bacteria and fungi in comparison to the control samples. The main strains of bacteria used are *Pseudomonas* spp. and *Bacillus* spp. and fungi—*Rhizobus* spp. Digestion of AG and PVA by microorganisms led to visible changes in the surface of the membranes after 30 and 60 days compared to the original membranes [32].

Biodegradation in time was also carried out with the use of activated sludge in membranes made of cellulose triacetate (CTA) and polyamide thin film composite (TFC). CTA membranes were found to be more resistant to biodegradation than TFC membranes. For both membranes, it was noticed that biodegradation caused an increase in pore size and the water and salt permeability. The results showed that CTA and TFC membranes may not be easily compatible with the membrane bioreactor [33]. Poly (dl-lactide-*co*-glycolide) (PLGA) membranes—were prepared by phase inversion and degraded under static culture conditions in 0.1 M PBS at 37 °C. After several weeks, loss of molecular weight and selective permeability was observed [34].

In this work, partially degradable PSF-PUR blend hollow fiber membranes were studied. PSF-PUR blend were used due to good miscibility of PSF and PUR and solubility of both polymers in the same used solvent (important from a technological point of view), the presence of ester bonds in PUR and well-known biocompatibility of PSF and PUR (potentially important in biomedical applications).The aim of this study was to obtain semipermeable HFMs using a polymer blend of PSF and synthesized PUR and evaluate the possibility of partial biodegradation by *Escherichia coli* bacteria of obtained membranes by assessing changes in transport-separation properties and morphology after the biodegradation process while maintaining a constant membrane cut-off point. Potentially, partial biodegradation would extend the useful life of the membranes. Received PURs, in their structure, contain ester bonds that potentially undergo biodegradation processes. Water hydrolysis is too slow, so it can be practically skipped in applications lasting up to several weeks/months. This is a confirmation of the hypothesis that polyesterurethanes as a membrane component undergo both alkaline hydrolysis [35] and biodegradation. Membrane transport-separation properties, morphology and biodegradation process with the use of *E. coli* cells were examined. *E. coli* is a Gram-negative, facultatively anaerobic, rod-shaped, coliform bacterium in natural environment found in the lower intestine of warm-blooded organisms. *E. coli* is frequently used as a model/representative organism in microbiology studies. Cultivated strains are well-adapted to the laboratory environment, and, unlike wild-type strains, have lost their ability to thrive in the intestine [36]. The biodegradation was carried out for 7 days, and then the membranes were re-evaluated for their transport-separation properties and changes in morphology. Transport-separation properties were estimated based on UFC and retention measurements, while morphologies were assessed using a scanning electron microscope (SEM).

## 2. Experimental

### 2.1. Hollow Fiber Membranes—Preparation and Characterization

#### 2.1.1. Materials

**Figure 1:**
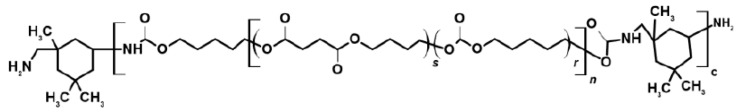
Structure of polyurethane (PUR).

**Figure 2:**
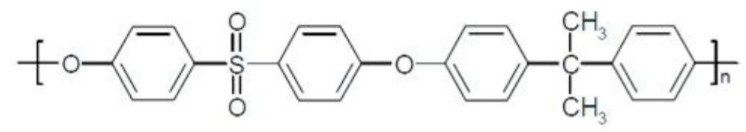
Structure of polysulfone (PSF).

Polysulfone (PSF) Udel 1700 NT LCD from Dow Corning(Midland, MI, USA), M.W. 70 kD; polyurethanes (PUR) with ester bonds in the structure (different % of ester bonds, marked as PUR 1 with ~80% of ester bonds, and PUR 2 with ~90% of ester bonds) (synthesized using methods from [37,38]; *N*-methyl-2-pyrrolidone (NMP) from Fluka(Buchs, Switzerland); *N*,*N*-Dimethylformamide (DMF) from Chempur(Karlsruhe, Germany), polyethylene glycols (PEGs) M.W. 4, 15 and 35 kD from Fluka, chicken egg albumin (CEA) M.W. 45 kD from Sigma-Aldrich(Munich, Germany), bovine serum albumin (BSA) M.W. 67 kD from Fluka and water 18.2 MΩ from MiliQ installation were used. Chemical structures of PUR and PSF are presented in Figure 1 and Figure 2.

#### 2.1.2. Hollow Fiber Membrane Preparation

Hollow fiber membranes were prepared using a dry/wet-spinning, phase inversion technique through extrusion of polymeric solution. The polymeric solution was prepared by dissolving PSF (concentrations: 27.0% (PSF_PUR-3), 24.0% (PSF-PUR-1 and PSF-PUR-4), 21.0% (PSF-PUR-2)) and PUR (concentrations: 2.5% (PSF_PUR-3), 5.00% (PSF-PUR-1 and PSF-PUR-4), 7.50% (PSF-PUR-2)) in two different flasks and stirring until complete dissolution. Then the solutions were mixed for 24 h. Table 1 provides information on the composition of the obtained membranes.

The membranes were obtained at the same spinning temperature 22 +/− 1 °C (gelling bath and casting solution), air gap 3–4 cm, relative humidity in the air gap 78–82%, core liquid pressure 110–120 mmHg, spinning speed 10–12 m/min to keep the reproducibility of the membranes. The detailed process of the membranes obtaining was described in our previous works [29,30,39,40]. Obtained membranes (20 capillaries, 5.8 cm long) were put into propylenes modules. Membranes were characterized and incubated with *E. coli* post-breeding medium for ~168 h. During biodegradation, the bacterial culture was kept in the stationary growth phase. After biodegradation the membranes were treated with 0.6% peracetic acid (for 24 h) and then with a 25% NaOCl solution (15 min). After treatment, demineralized water was passed through the membrane’s walls for removing peracetic acid and NaOCl and clearing the membranes. Control samples were treated with demineralized water for~168 h, then treated with 0.6% peracetic acid (for 24 h) and after that with a 25% NaOCl solution (15 min).

#### 2.1.3. Membrane Characterization

Membranes were characterized twice—before and after biodegradation.

- Ultrafiltration Coefficient

The ultrafiltration coefficient (*UFC*) was calculated according to the formula:

(1) $$UFC=\frac{v}{p\cdot t\cdot a}$$

where: *v*—the volume of pure water [cm^3^]; *p*—transmembrane pressure [hPa]; *t*—the time of measure [min]; *a*—nominal membrane’s area in a module [m^2^].

The hydraulic permeability was measured as a volume of pure water passed through the membrane’s walls during the period of established time [min] and under established transmembrane pressure [hPa].

- Retention Measurements and Cut-Off Evaluation

The membrane retention [%] was defined as:

(2) $$R=\left\{1-\left(\frac{C_{P}}{C_{F}}\right)\right\}\times 100\%$$

where: *R*—retention coefficient; *C_P_*—concentration of marker in permeate [g/dm^3^]; *C_F_*—concentration of marker in the feed [g/dm^3^].

The concentrations of markers (1 g/dm^3^) were evaluated by a UV-spectrophotometer (HITACHI U-3010) at 280 nm for CEA 45 kD and BSA 67 kD and 190 nm wavelength for PEGs: 4, 15 and 35 kD.

- SEM

Morphology of the membranes before and after biodegradation was characterized by SEM using the Hitachi TM-1000 microscope. The membranes were cut in liquid nitrogen, dried and then coated with a 10-nm gold layer, using a sputtering device (EMITECH K 550 X).

- Evaluation of Pores by Computer Analysis of SEM Images–MeMoExplorer^TM^ Software

For the analysis, the 15 SEM photomicrographs of membrane’s cross-section before (denoted by 1 for PSF-PUR-1, 2 for PSF-PUR-2, 3 for PSF-PUR-3, 4 for PSF-PUR-4) and after biodegradation (denoted by 1.2 B, 1.3 B, 1.4 B, 2.2 B, 2.3 B, 2.4 B, 3.2 B, 3.3 B, 3.4 B, 4.2 B, 4.3 B, 4.4 B), and control samples (denoted by 1.1„0”, 2.1„0”, 3.1„0”, 4.1„0”) were taken according to the description in Section 2.1.3. SEM images were taken with the same size with a microscope magnification of x1000. Then they were analyzed by the MeMoExplorer^TM^ Software which involved contouring of pores and measurement of their surfaces. Additionally, the MemoExplorer^TM^ Software is able to partition pores into 8 size–classes (Table 2) and measurement of total areas (porosity coefficients).

The received data were processed into statistical parameters like average (Ave), standard deviation (SD), instability coefficients (SD/Ave).

- FT-IR

FT-IR spectra were recorded on a Nicolet iS5 Mid Infrared FT-IR (resolution = 4 cm^−1^, number of scans = 32, type of crystal used=diamond). Spectrometer equipped with iD7 ATR Optical Base.

### 2.2. Membranes Biodegradation

#### 2.2.1. Materials

Bacterial strain—*Escherichia coli* ATCC 8739. Growth media—Davis Minimal Broth without Dextrose from Becton Dickinson (BD); Tryptic Soy Broth (TSB) and Tryptic Soy Agar from (TSA) BIOCORP; 0.85% sodium chloride (Merck Millipore).

#### 2.2.2. Storage and Handling of *E. coli* Glycerol Stock

Bacteria were grown in liquid TSB medium for 24 h, 30 °C, 250 rpm. Subsequently, glycerol was added to the final concentration of 20%. The mixture was portioned and frozen at −80 °C.

#### 2.2.3. *E. coli* Culture Growth and Membranes Biodegradation

The *E. coli* culture was carried out in 1 L flow bioreactor in 600 cm^3^ of Davis Minimal Broth without Dextrose, inoculated by the addition of thawed 600 µL of earlier prepared bacterial glycerol stock. The cultures were grown at room temperature (about 22–25 °C) with stirring.

Twice a day about 30 mL of a fresh medium was added to the culture with simultaneous draining of the same portion of the post-culture fluid with bacterial cells to the membrane module. The medium exchange was carried out in each case within 45 min using a peristaltic pump with a flow rate of 0.6 mL/min.

After degradation, the module membranes were treated for 24 h with a 0.6% peracetic acid solution to remove the biofilm formed and then treated with water to remove the acid (pH controlled). To ensure that all biofilm was removed or to remove its remnants, the membranes were treated for 15 min with 25% (*w*/*w*) NaOCl solution (25 g of the solution was diluted in 75 g DI water). The degradation was performed for four membranes, each in triplicate.

Control modules were also performed—DI water was passed through one module for 7 days, and then the biodegradable modules were treated with peracetic acid and NaOCl solution to check that peracetic acid and NaOCl did not affect membrane degradation.

#### 2.2.4. Measurement of Cell Density and Viability

During biodegradation, the bacterial growth was monitored daily by spectrophotometric measurement at 600 nm (OD_600_).

The viability of *E. coli* was measured at the beginning and end of each culture. For this purpose series of 10-fold dilutions of the culture fluid in the 0.85% sodium chloride solution were prepared. The 10^−6^, 10^−5^, 10^−4^ dilutions were plated on TSA medium and after 24 h incubation (30 °C) colonies were counted. The final viability result is given as the quantity of CFU/mL.

## 3. Results and Discussion

### 3.1. Membranes Characterization

#### 3.1.1. Ultrafiltration Coefficient

Table 3 presents the changes in UFC for membranes before and after biodegradation. Module 1 (marked as control) for all of the membranes was treated with water for 1 week and after that with 0.6% peracetic acid and 25% NaOCl solution and used as a control to prove the absence of peracetic acid and NaOCl effects on membrane properties.

The obtained UFC values indicated a clear influence of biodegradation on changes in the transport-separation properties of membranes. There was no significant change between the UFC_b + a_ and UFC_NaOCl_, which meant that the peracetic acid successfully destroyed the biofilm formed by the *E. coli*. For the modules marked as 1, there was no significant increase or decrease in UFC, UFC_a_ and UFC_NaOCl_ values in any of the membranes (differences similar to measurement errors), which proved the lack of influence of peracetic acid and NaOCl on the UFC change.

The module 1 UFC of the PSF-PUR-1 membrane after treatment with peracetic acid and NaOCl solution did not change its value, indicating that peracetic acid and NaOCl did not affect the transport-separation properties of the membrane. For modules 2, 3 and 4 that were treated with *E. coli*, increases in $\frac{{\mathrm{UFC}}_{\mathrm{NaOCl}}}{{\mathrm{UFC}}_{0}}$ ranging from 2.4 to 2.8 were recorded.

For the PSF-PUR-2 membrane, also in the case of the control test, there were no changes in the UFC values after treatment with peracetic acid and NaOCl solution. In contrast, increases in $\frac{{\mathrm{UFC}}_{\mathrm{NaOCl}}}{{\mathrm{UFC}}_{0}}$ for modules 2, 3, 4 ranged from 5.5 to almost 8.8.

PSF-PUR-3 membrane module 1 also had no change in UFC values. The ratios of $\frac{{\mathrm{UFC}}_{\mathrm{NaOCl}}}{{\mathrm{UFC}}_{0}}$ modules 2, 3, 4 are very stable and were between 1.3 and 1.7.

The PSF-PUR-4 membrane control test module, as well as all control modules, showed no changes in the UFC value after treatment with peracetic acid and NaOCl solution. Module 2 showed the smallest change in UFC after biodegradation and the ratio $\frac{{\mathrm{UFC}}_{\mathrm{NaOCl}}}{{\mathrm{UFC}}_{0}}$ was less than 1.3. On the other hand, modules 3 and 4 already achieved an almost threefold increase, 2.8 and 2.7, respectively.

Changes in the UFC values after biodegradation indicated the effect of biodegradation on the transport-separation properties of membranes treated with *E. coli*. The highest increases were recorded for the PSF-PUR-2 membrane and the lowest for PSF-PUR-3. For PSF-PUR-1 and PSF-PUR-4 membranes (except for module 2) the increases in UFC were very similar. The observation was that the increase in UFC after biodegradation may have been dependent on the weight content of PUR in the membrane (UFC values cannot be equal for PSF-PUR-1 and PSF-PUR-4 since the membranes were different due to usage of different solvent and type of PUR). The PSF-PUR-2 membrane, with the highest increase, contained 30% by weight of PUR, PSF-PUR-1 and PSF-PUR-4 membranes, for which similar increases were recorded (UFC changes were recorded as being between the highest and the lowest ratio $\frac{{\mathrm{UFC}}_{\mathrm{NaOCl}}}{{\mathrm{UFC}}_{0}}$ contained 20 wt.% PUR, while the PSF-PUR-3 membrane contained only 10 wt.% PUR. Comparing the UFC with the literature data, the expected results were obtained, confirming that the degradation of membranes (hydrolysis or biodegradation) caused an increase in the UFC value [29,30,33,35,39,41].

#### 3.1.2. Retention and Cut-Off Measurement

**Figure 3:**
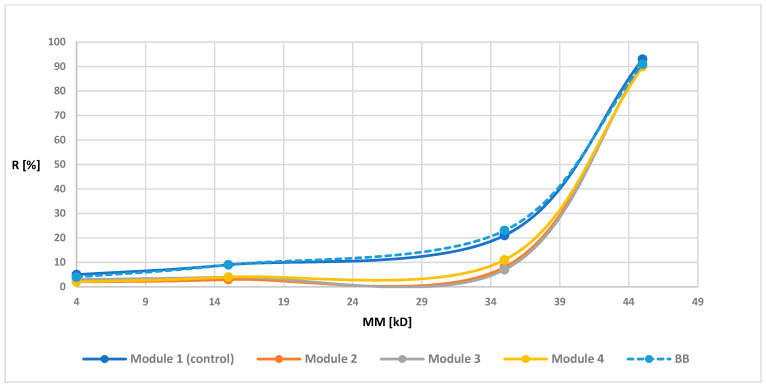
Retention coefficient values for different markers for PSF-PUR-1 membrane before biodegradation (BB), after biodegradation (module 2, module 3, module 4) and control (module 1).

**Figure 4:**
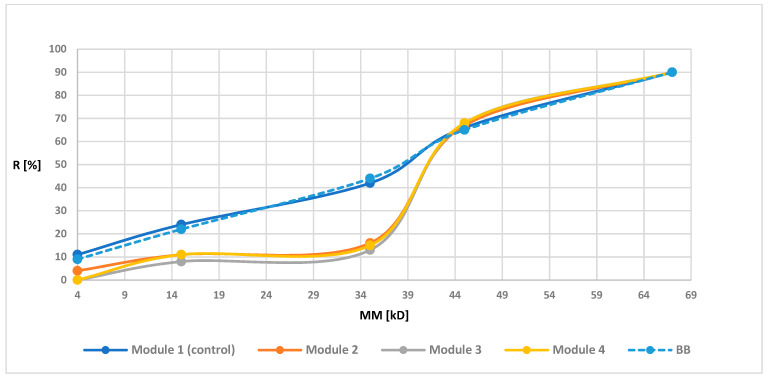
Retention coefficient values for different markers for PSF-PUR-2 membrane before biodegradation (BB), after biodegradation (module 2, module 3, Module 4) and control (module 1).

**Figure 5:**
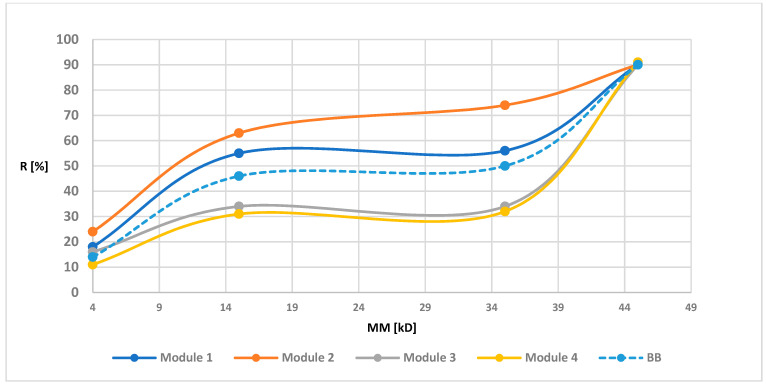
Retention coefficient values for different markers for PSF-PUR-3 membrane before biodegradation (BB), after biodegradation (module 2, module 3, module 4) and control (module 1).

**Figure 6:**
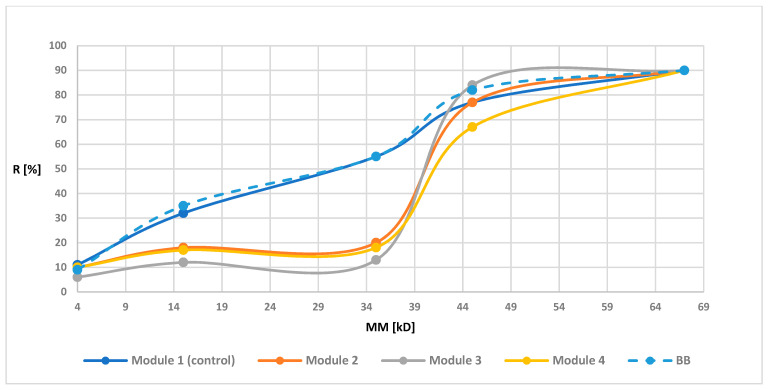
Retention coefficient values for different markers for PSF-PUR-4 membrane before biodegradation (BB), after biodegradation (module 2, module 3, module 4) and control (module 1).

Retention measurements were prepared for all modules before and after the biodegradation. The Figure 3, Figure 4, Figure 5 and Figure 6 of the relationship between the degree of retention and the molar mass of the marker are presented below.

In order to illustrate the retention changes for membranes after the biodegradation process more precisely, in the graphs of the retention dependence on the molar mass of the markers, an additional data series was added for the membrane before the biodegradation process, marked as “before biodegradation (BB)” (different line style in the diagram is additionally used). Module 1 (control modules) for all tested membranes had a similar R-value to the BB. These values were not identical since the BB membrane contained the average retention values from four modules, while the values shown for module 1 were values obtained from only one module. However, the obtained results indicated (as in the case of maintaining similar UFC values for the control samples) the lack of influence of peracetic acid and NaOCl on the transport-separation properties of the membrane. On the other hand, in the case of biodegradable modules, the obtained dependence of the percentage of retention on the molar mass of the marker (combined with an increase in the porosity coefficient presented in 3.1.4) indicated (mostly visible for membranes PSF-PUR-2 and PSF-PUR-4) an improvement in the selectivity of membranes after biodegradation (except module 2 for the PSF-PUR-3 membrane, where after biodegradation an increase in the value of retention coefficients was noticeable but significantly different from the values for membranes before biodegradation and this type of discrepancy can be considered as an acceptable error limit). Changes in the values of retention coefficients may be caused by changes in the structure of the membrane occurring as a result of the biodegradation process. The removal of PUR polymer may cause new inner canal openings inside the membrane structure due to breakdown of ester bonds. Comparing the PSF-PUR membrane to the CTA membrane, it was noticed that biodegradation in the case of PSF-PUR membranes caused an increase in selectivity, in contrast to the CTA membranes [33]. However, comparing the method of polymer degradation (hydrolysis versus biodegradation) for the studied membranes, the value of the retention coefficient after biodegradation decreased, as in the case of the hydrolysis of PSF-CA membranes with a2 M NaOH solution [30]. However, biodegradable membranes showed greater selectivity compared to membranes made of the same PUR polymer undergoing with a 1 M NaOH solution [35], which may indicate a better impact of biodegradation on the transport-separation properties of the membrane.

#### 3.1.3. SEM Analysis

**Figure 7:**
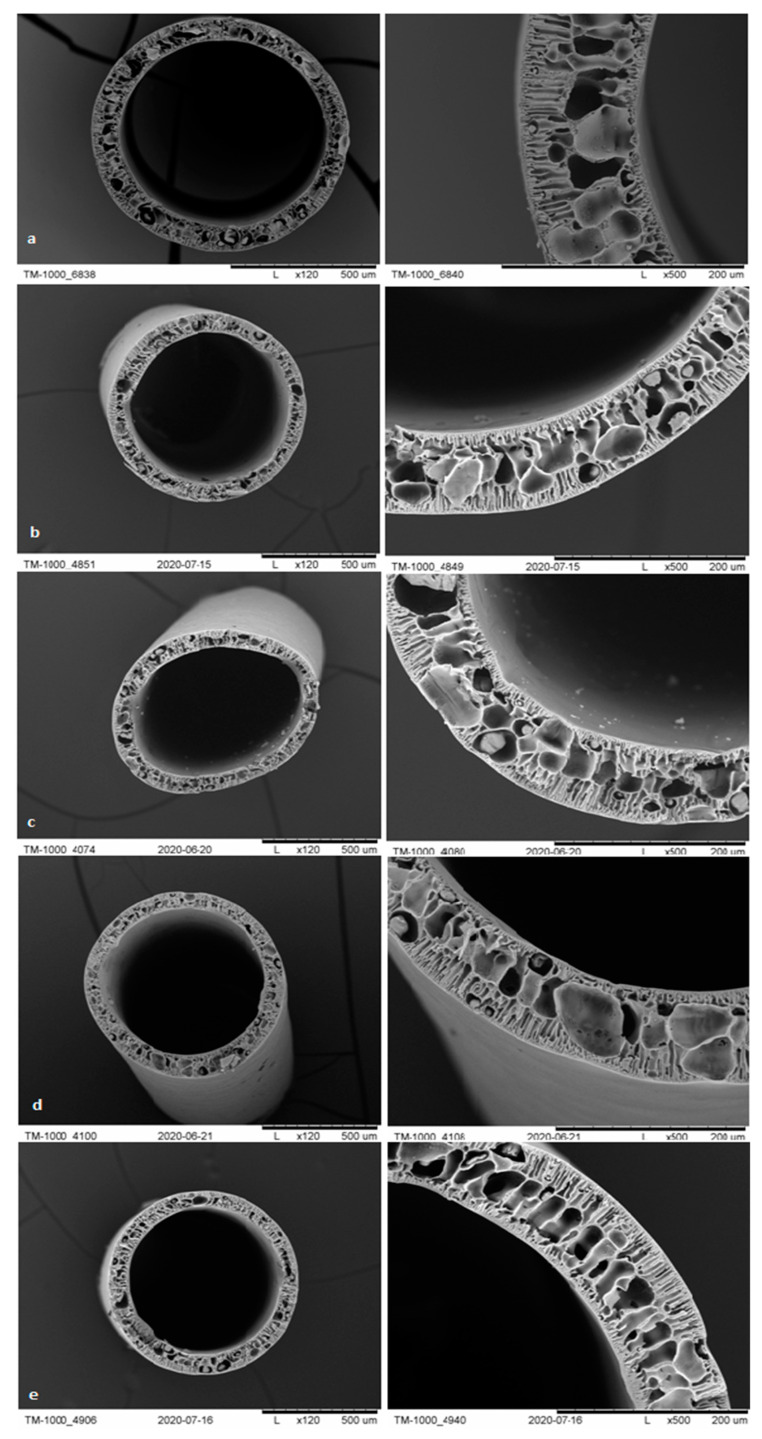
Cross-section and fragment of the PSF-PUR-1 before biodegradation (**a**), and module 1 (control) (**b**), module 2 (**c**), module 3 (**d**), module 4 (**e**) after biodegradation.

**Figure 8:**
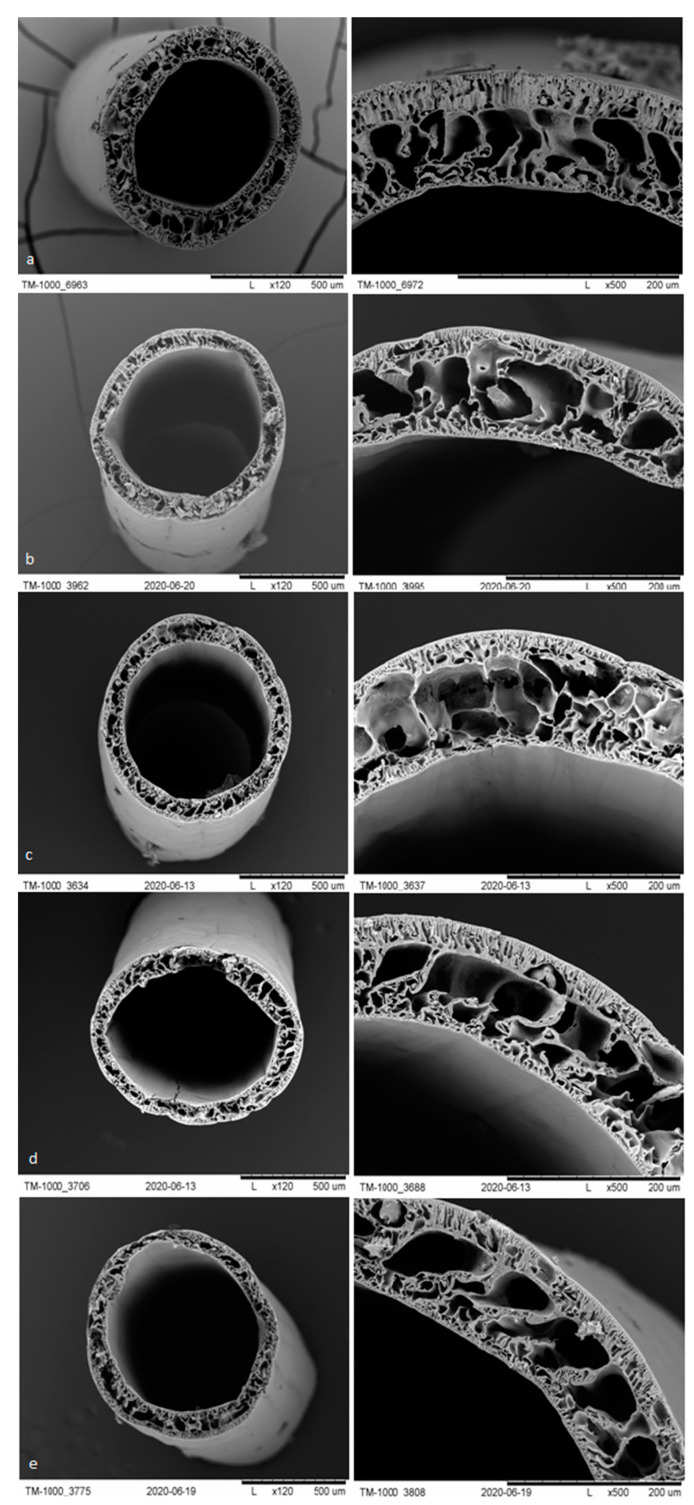
Cross-section and fragment of the PSF-PUR-2 before biodegradation (**a**), and: module 1 (control) (**b**), module 2 (**c**), module 3 (**d**), module 4 (**e**) after biodegradation.

**Figure 9:**
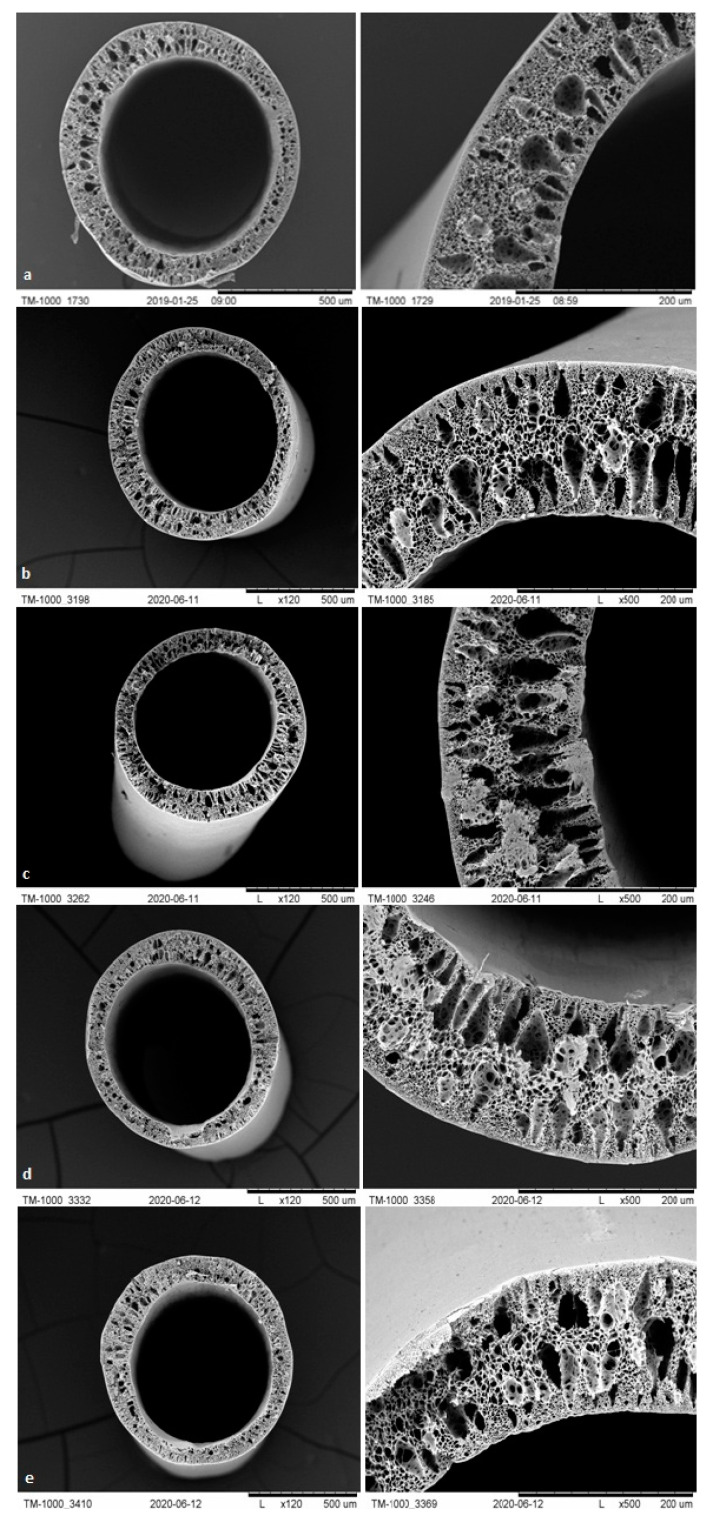
Cross-section and fragment of the PSF-PUR-3 before biodegradation (**a**), and module 1 (control) (**b**), module 2 (**c**), module 3 (**d**), module 4 (**e**) after biodegradation.

**Figure 10:**
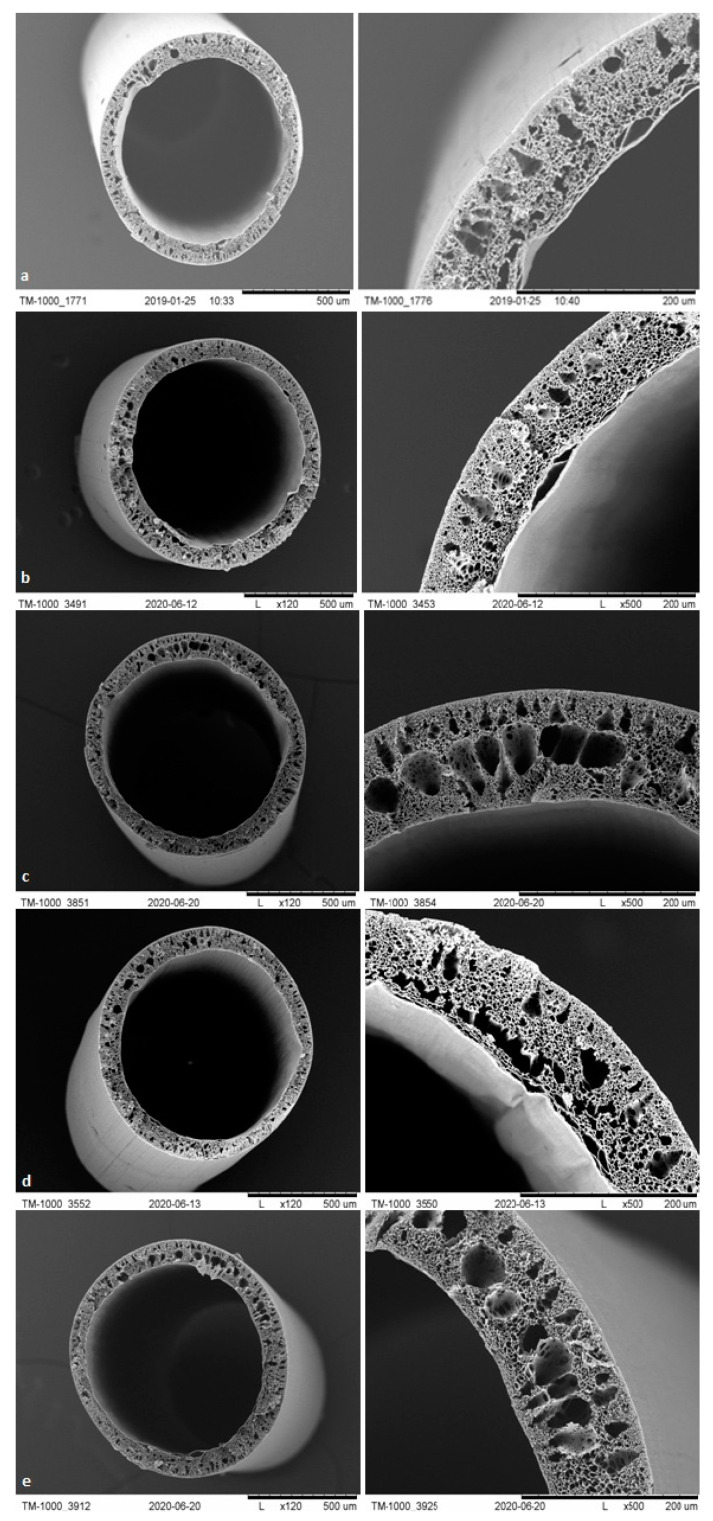
Cross-section and fragment of the PSF-PUR-4 before biodegradation (**a**), and module 1 (control) (**b**), module 2 (**c**), module 3 (**d**), module 4 (**e**) after biodegradation.

The membranes from each module after biodegradation were subjected to a separate morphological assessment using SEM. Figure 7, Figure 8, Figure 9 and Figure 10 show cross-sections and fragments of membranes before biodegradation and of membranes from each module after biodegradation.

Changes in morphology after the biodegradation process were noticeable. No changes in skin-layer but larger surface pores in epidermal layer were noticeable for modules after biodegradation. In some cases (PSF-PUR-2), the resulting pores could be classified as macropores. However, the assessment of changes by the human eye was not precise and accurate—it was impossible to make a reliable assessment of changes in pore size after the biodegradation process.

#### 3.1.4. Evaluation of Pore Distributions of Membranes Using MeMoExplorer^TM^ Software

The size of the pores was analyzed based on SEM images of membranes before and after biodegradation. The area of pores in eight size-classes is presented in Figure 9. The results show the average percentage of appropriate pore size in relation to the whole SEM photomicrographs size.

Where: PSF-PUR-1, PSF-PUR-2, PSF-PUR-3, PSF-PUR-4—area of pores for membranes before biodegradation, 1.1„0”; 2.1„0”, 3.1„0”, 4.1„0”—area of pores for controls of PSF-PUR-1, PSF-PUR-2, PSF-PUR-3, PSF-PUR-4 membranes after treatment with peracetic acid and NaOCl, 1.2 B, 1.3 B, 1.4 B, 2.2 B, 2.3 B, 2.4 B, 3.2 B, 3.3 B, 3.4 B, 4.2 B, 4.3 B, 4.4 B—area of pores for modules of PSF-PUR-1, PSF-PUR-2, PSF-PUR-3, PSF-PUR-4 membranes after biodegradation and treatment with peracetic acid and NaOCl.

**Figure 11:**
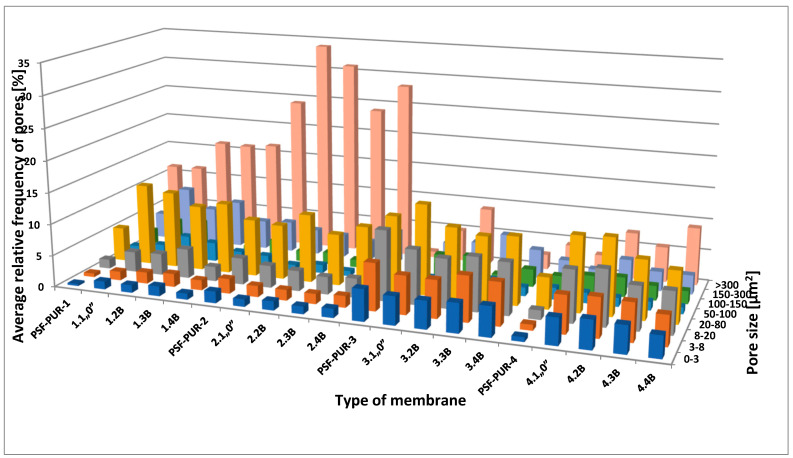
The average relative frequency of pores in eight size–classes for every membrane.

Figure 11 shows the mean of the pores in eight categories of pore-size for each membrane. The outcomes are expressed as a mean of percentage. The largest sizes of pores were noticed in membrane PSF-PUR-2, in particular in size-classes of 8. The percentage of pore sizes >300 µm^2^ was as high as 33.97%. It is noteworthy that membrane PSF-PUR-2 had the highest proportion of PUR (the PSF:PUR ratio was 7:3). This could possibly have had an impact on the structure and hence the pore size. On the other hand, in membrane PSF-PUR-4, the smallest percent of pores were noticed, especially for the size-class category of 7 where 4.45% was not exceeded.

**Figure 12:**
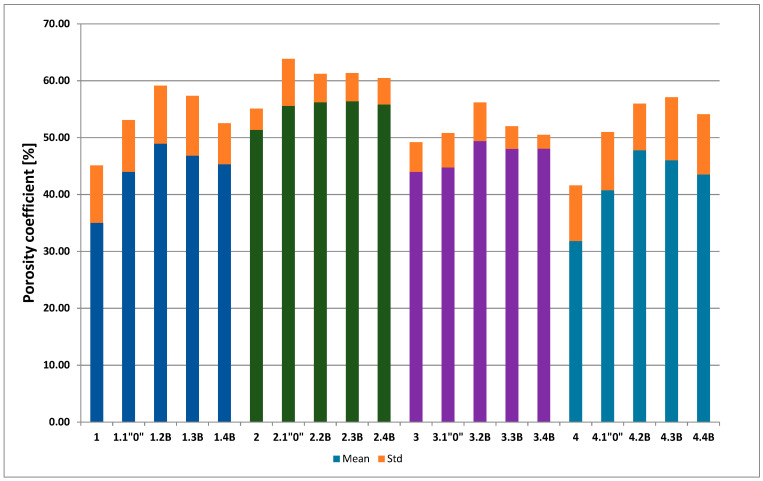
Percent of porosity coefficients [%] before (1, 2, 3, 4) and after biodegradation (B), including control samples (“0”).

**Figure 13:**
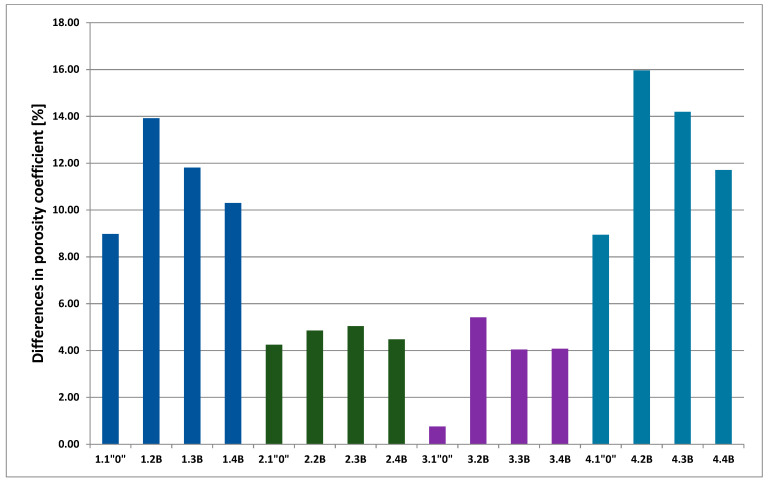
The differences in porosity coefficient [%] of membranes before and after biodegradation (control samples included).

Figure 12 shows the percentage of the total number of pores (porosity coefficients) for each membrane. As can be seen, the highest value was for membrane PSF-PUR-2.4 B and was almost 55%. The smallest value was for membrane PSF-PUR-4 and it was about 31% where even after biodegradation the magnitude was below 50%. Additionally, biodegradation should be looked at in more detail. Therefore, it is presented in a separate diagram, as shown in Figure 13. The biggest difference in porosity coefficient was noticed for membranes PSF-PUR-1 and PSF-PUR-4. The pores, after biodegradation, were enlarged respectively by almost 14% (for PSF-PUR-1) and 16% (for PSF-PUR-4). It is worth paying attention to the equal PUR content in membranes, where its ratio to PSF was 8:2 and despite the differences in type of solvent and PUR used, the increase of pores were similar for these membranes. The lowest difference in the porosity coefficient was for membranes PSF-PUR-2 and PSF-PUR-3. It was at a similar level, from 4% to 5%. As it can be seen, treatment with peracetic acid and NaOCl solution may have influenced the membrane porosity ((control module of PSF-PUR-2 (2.1”0”) exhibited a similar difference in porosity with modules that underwent biodegradation and followed with peracetic acid and NaOCl treatment (2.2 B–2.4 B)—thus it can be assumed that in general biodegradation had the smallest impact on the changes in porosity in the case of this membrane. Comparing UFC and retention coefficient values it can be seen that for membranes where the treatment with peracetic acid and NaOCl solution may have influenced porosity of a membrane, only biodegradation process had significant influence on transport-separation properties. According to the results it can be noticed that an important factor, apart from the PUR content, was the distribution of polymers in the membrane structure (depended on: membrane composition, air gap, accuracy of mixing and dissolution time), which is important in further membrane processes/fates. We had no influence at this stage of its preparation.

**Figure 14:**
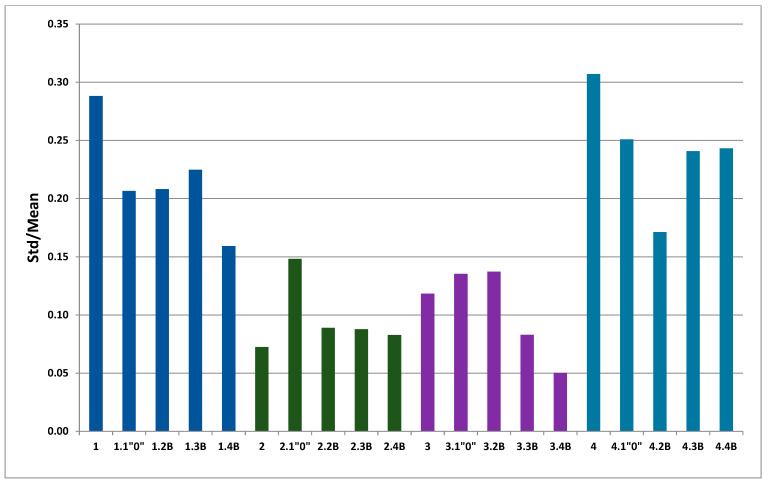
The instability coefficients of membranes, before and after biodegradation.

Besides, computer analysis of data from MeMoExplorer^TM^ could evaluate the reproducibility of obtaining parameters in membranes production process (instability coefficient). It was calculated by the ratio of standard deviation (SD) of the porosity coefficient to averaged value (Ave) of the porosity coefficient of membranes. The pore repeatability was also studied after biodegradation. Coefficients of dissimilarity, before and after biodegradation for each membrane are presented in Figure 14. Membrane PSF-PUR-2 appeared to be the best one from the stability point of view. The instability coefficient was the smallest for this sample and it was only about 0.07. After biodegradation, the best result was noticed for PSF-PUR-3.4 B, and the value was about 0.05. A stabilizing effect was observed in each case for both membranes, before and after biodegradation (and also control samples), indicating the repeatability of the pore size. It was generally low and did not exceed 0.31.

Referring to Figure 12, Figure 13 and Figure 14, the analysis of SEM images using MeMoExplorer^TM^ Software showed that after biodegradation the porosity of the membranes increases. Comparing the data for the controls and biodegradable modules, there was a noticeable difference in porosity percentages in favor of membranes treated with *E. coli* bacteria. Undoubtedly, the influence of peracetic acid and a NaOCl solution could have had an effect on a slight increase in porosity of the control samples, however, the treatment with *E. coli* clearly influenced the higher values for the modules after biodegradation (marked as numbers 2, 3, 4 for all analyzed membranes).It was expected and confirmed, that after biodegradation [33] and chemical degradation (hydrolysis) [29,30,35,40] the size of pores increased due to partial degradation of PUR.

#### 3.1.5. FT-IR Analysis

FT-IR spectra were recorded for all biodegradable membrane modules: PSF-PUR-1, PSF-PUR-2, PSF-PUR-3, PSF-PUR-4 and for the same membranes before biodegradation. The comparison of changes in the peaks corresponding to wavelengths in the ranges 1760–1730 and 1700–1630 cm^−1^ is presented in Table 4, Table 5, Table 6 and Table 7. The table is divided into two columns, in the column “Module” the numbers of modules after biodegradation are marked with numbers, and additionally, a row was added for each membrane. “Before biodegradation”, which includes the fragment of the spectrum for the above-mentioned range recorded for the membrane before biodegradation.

The 1760–1730 cm^−1^ peak is assigned to the C=O functional group derived from ester bonds, while 1700–1630 cm^−1^ can be attributed to the amine and carbamate groups. Although the FT-IR technique is not a method for quantification, it perfectly illustrated the changes that occurred in the membrane after the biodegradation process and proved that during biodegradation, the ester or carbamate bonds contained in the polyurethane structure were broken down. The disappearance of the peaks for both functional groups led to the conclusion that PUR biodegradation took place through several mechanisms simultaneously: the breakdown of ester bonds or carbamates. Undoubtedly, greater changes were observed for the amino and carbamate groups, which may lead to the conclusion that biodegradation occurred to a greater extent at the ends of the polymer chain (according to the PUR structure, in which the amino and carbamate groups are present at the ends of the polymer chains). However, the obtained results may suggest that the bacteria treated the entire polymer as nutrition substrate, which was observed in changes in FT-IR spectra and transport-separation properties of the membranes.

### 3.2. Membrane Biodegradation

**Figure 15:**
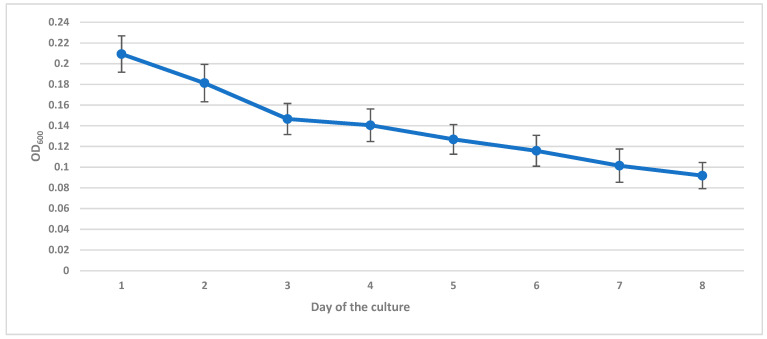
Daily monitoring of the cultures OD_600_ changes.

The culture condition and cell density were monitored daily by measuring the absorbance of the post-culture fluid (Figure 15), simultaneously on the first and last day of culture the cells were plated on TSA medium assessing their viability. The results were presented as averaging measurements from all cultures. The cultures were in the stationary growth phase. Slight deviations between measurements suggest that the conditions between individual experiments were stable. Maintaining the culture in the stationary growth phase was important due to the presence of metabolites secreted into the medium, which may also affect membrane stability. Between the first and last days of the cultures, some decline in the viability of *E. coli* was observed (5.45∙10^8^ ± 7.76∙10^7^ CFU/mL vs. 1.66·10^8^ ± 4.99∙10^7^ CFU/mL). However, the viability of cells on the eighth day still reached the order of 10^8^, which indicated that the state of the culture allowed for efficient biodegradation of membranes.

## 4. Conclusions

It was found that all tested membranes (modules 2,3,4 for each membranes) were partially biodegradable due to changes in transport-separation properties, selectivity and increase of porosity coefficients. It was possible to obtain the repeatability of the experiment due to providing similar biodegradation conditions for all modules. Changes in the selectivity of membranes after the biodegradation process are noticeable, which is not affected by the action of peracetic acid and sodium hypochlorite, only the biodegradation process improves the selectivity of the membranes. For all modules after biodegradation, a change in the dependence of retention on the marker molar mass is noticeable. Biodegradation may improve the selectivity of membranes against individual markers without changing the cut-off point established for the membrane before biodegradation. The analysis of the SEM images with the MeMoExplorer^TM^ Software showed changes in the porosity of the membranes after biodegradation. It is another method that allows to determine and confirm the influence of *E. coli* bacteria. It was found that washing the membranes with both peracetic acid solution and NaOCl solution did not affect the filtration parameters of the membranes but may affect their morphology. This applies to contact not exceeding 24 h for peracetic acid and 15–20 min for NaOCl solution. This proves that these membranes probably can be regenerated and sterilized with both of these solutions. It has been reported that CTA membranes may not be very compatible and may not necessarily be used for biodegradation processes [33]. On the other hand, it is believed that PSF-PUR membranes can be used in bioprocesses due to the improved selectivity after the degradation process, which may be due to the presence of a stable polymer in the membrane structure next to the partially degradable polymer. The authors hope that two-component membranes that are partially biodegradable, can be used for macroencapsulation of biologically active agents implanted subcutaneously, intramuscularly and even intraperitoneally, in membrane bioreactors in cell cultures, as a solution for the separation of bio-seam products in bioreactors, e.g., in the production of lactic acid or perhaps as medical applications (production of sera and vaccines)due to current research trends searching for new polymeric membranes with well-defined morphology and structure, especially with controlled porosity. Higher UFC value, increased selectivity, higher porosity may affect the duration of the membrane process in cases where regeneration or replacement of membranes during the process is often impossible or very complicated. Due to partial biodegradation, it would also be possible to partially compensate for the fouling phenomenon and potentially PUR can be taken into consideration as a pore precursor instead of PEG/PVP. It can be said that biodegradation is a better method of degradation of PSF-PUR membranes compared to hydrolysis due to the fact that the selectivity of the membranes was improved after biodegradation [35].

## Figures and Tables

**Table 1:** Composition of the membranes.

Membrane	PUR	PSF:PUR Weight Ratio	Solvent
PSF-PUR-1	PUR 1	8:2	NMP
PSF-PUR-2		7:3	
PSF-PUR-3	PUR 2	9:1	DMF
PSF-PUR-4		8:2	

**Table 2:** Size–classes of pores.

Size-Classes	1	2	3	4	5	6	7	8	9
Size [μm^2^]	0÷3	3÷8	8÷20	20÷80	80÷100	100÷150	150÷300	>300	Total (porosity coefficients)

**Table 3:** Ultrafiltration coefficients for all modules before (UFC_0_) and after biodegradation (UFC_b + a_, UFC_NaOCl_).

	UFC_0_ $\left[\frac{cm^{3}}{min\cdot m^{2}\cdot hPa}\right]$	UFC_a+b_ $\left[\frac{cm^{3}}{min\cdot m^{2}\cdot hPa}\right]$	UFC_NaOCl_ $\left[\frac{cm^{3}}{min\cdot m^{2}\cdot hPa}\right]$	$\frac{UFC_{NaOCl}}{UFC_{0}}$
**PSF-PUR-1**				
Module 1 (control)	12.188	12.070 (UFC_a_)	12.109	0.994
Module 2	6.784	17.100	17.007	2.507
Module 3	10.897	30.897	30.897	2.835
Module 4	7.654	18.769	18.712	2.445
PSF-PUR-2				
Module 1 (control)	2.390	2.430 (UFC_a_)	2.430	1.017
Module 2	2.641	18.343	18.363	6.953
Module 3	3.239	17.829	17.923	5.533
Module 4	2.204	19.274	19.391	8.798
PSF-PUR-3				
Module 1 (control)	0.345	0.342 (UFC_a_)	0.340	0.985
Module 2	0.355	0.469	0.469	1.321
Module 3	0.302	0.407	0.407	1.348
Module 4	0.461	0.767	0.767	1.664
PSF-PUR-4				
Module 1 (control)	0.0268	0.0274 (UFC_a_)	0.0281	1.0485
Module 2	0.0537	0.0685	0.0694	1.2924
Module 3	0.0406	0.1052	0.1140	2.8079
Module 4	0.0232	0.0611	0.0629	2.7112

Where: UFC_0_—UFC before biodegradation, UFC_b + a_—UFC after biodegradation and treatment with peracetic acid ((UFC_a_) = UFC for control modules that were treated with water and after 7 days with peracetic acid), UFC_NaOCl_—UFC_b + a_/(UFC_a_) after treatment with NaOCl.

**Table 4:** Comparison of FT-IR spectra before and after biodegradation for PSF-PUR-1 membrane.

Module	Fragment of the FTIR Spectrum [cm^−1^]
Before biodegradation	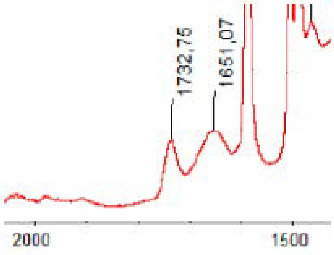
1. control	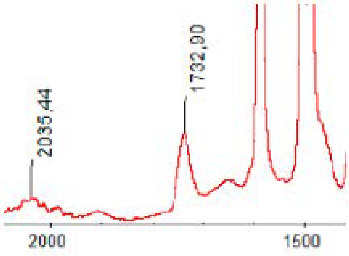
2. after biodegradation	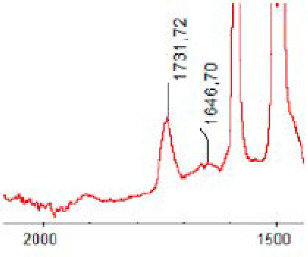
3. after biodegradation	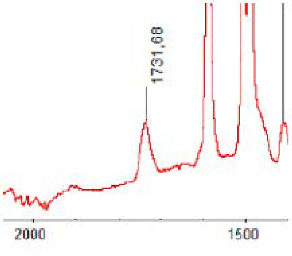
4. after biodegradation	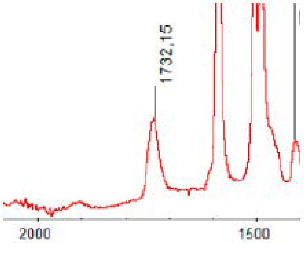

**Table 5:** Comparison of FT-IR spectra before and after biodegradation for PSF-PUR-2 membrane.

Module	Fragment of the FTIR Spectrum [cm^−1^]
Before biodegradation	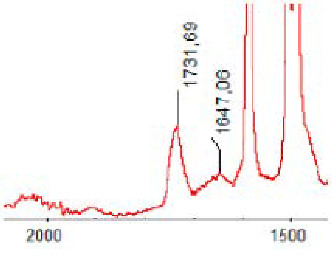
1. control	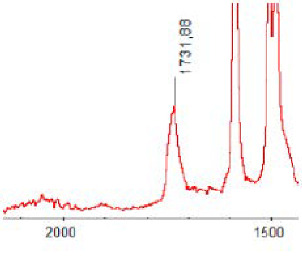
2. after biodegradation	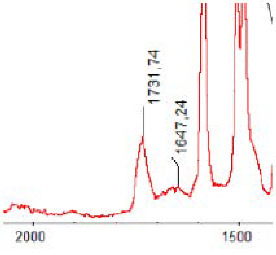
3. after biodegradation	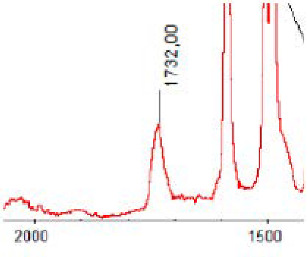
4. after biodegradation	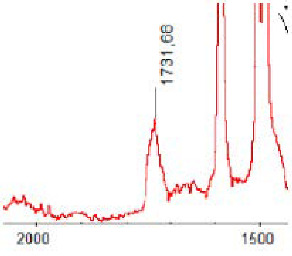

**Table 6:** Comparison of FT-IR spectra before and after biodegradation for PSF-PUR-3 membrane.

Module	Fragment of the FTIR Spectrum [cm^−1^]
Before biodegradation	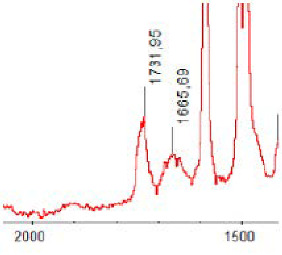
1. control	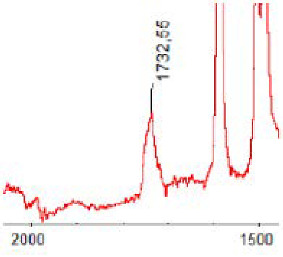
2. after biodegradation	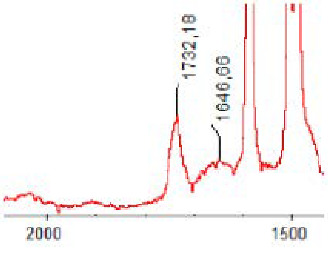
3. after biodegradation	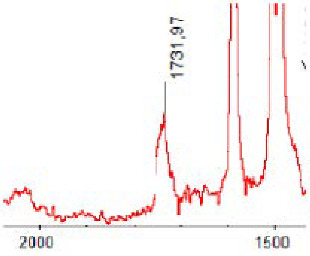
4. after biodegradation	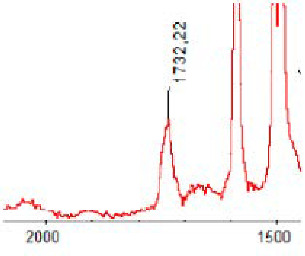

**Table 7:** Comparison of FT-IR spectra before and after biodegradation for PSF-PUR-4 membrane.

Module	Fragment of the FTIR Spectrum [cm^−1^]
Before biodegradation	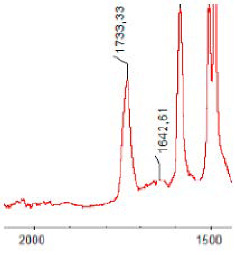
1. control	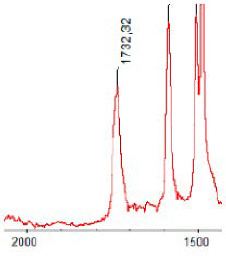
2. after biodegradation	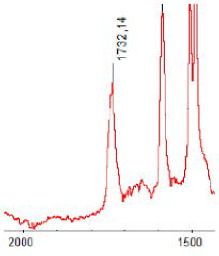
3. after biodegradation	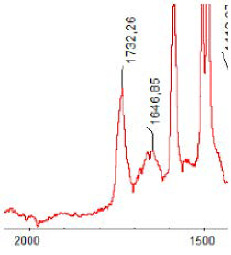
4. after biodegradation	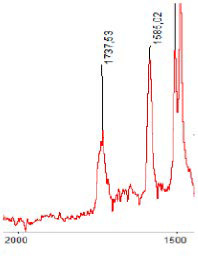

## Data Availability

Not applicable.
